# A novel expression platform for the production of diabetes-associated autoantigen human glutamic acid decarboxylase (hGAD65)

**DOI:** 10.1186/1472-6750-8-87

**Published:** 2008-11-17

**Authors:** Xiaofeng Wang, Martin Brandsma, Reynald Tremblay, Denis Maxwell, Anthony M Jevnikar, Norm Huner, Shengwu Ma

**Affiliations:** 1 Department of Biology, University of Western Ontario, London, Ontario, N6A 5B7, Canada; 2 Transplantation Immunology Group, Lawson Health Research Institute, London, Ontario, N6A 4G5, Canada; 3 Plantigen Inc., 700 Collip Circle, London, Ontario, N6G 4X8, Canada; 4 The Biotron Centre for Experimental Climate Change, The University of Western Ontario, 1151 Richmond Street N., Ste. 5150 SSB, London, Ontario, N6A 3K7, Canada

## Abstract

**Background:**

Human glutamic acid decarboxylase 65 (hGAD65) is a key autoantigen in type 1 diabetes, having much potential as an important marker for the prediction and diagnosis of type 1 diabetes, and for the development of novel antigen-specific therapies for the treatment of type 1 diabetes. However, recombinant production of hGAD65 using conventional bacterial or mammalian cell culture-based expression systems or nuclear transformed plants is limited by low yield and low efficiency. Chloroplast transformation of the unicellular eukaryotic alga *Chlamydomonas reinhardtii *may offer a potential solution.

**Results:**

A DNA cassette encoding full-length *hGAD65*, under the control of the *C. reinhardtii *chloroplast *rbc*L promoter and 5'- and 3'-UTRs, was constructed and introduced into the chloroplast genome of *C. reinhardtii *by particle bombardment. Integration of *hGAD65 *DNA into the algal chloroplast genome was confirmed by PCR. Transcriptional expression of *hGAD65 *was demonstrated by RT-PCR. Immunoblotting verified the expression and accumulation of the recombinant protein. The antigenicity of algal-derived hGAD65 was demonstrated with its immunoreactivity to diabetic sera by ELISA and by its ability to induce proliferation of spleen cells from NOD mice. Recombinant hGAD65 accumulated in transgenic algae, accounts for approximately 0.25–0.3% of its total soluble protein.

**Conclusion:**

Our results demonstrate the potential value of *C. reinhardtii *chloroplasts as a novel platform for rapid mass production of immunologically active hGAD65. This demonstration opens the future possibility for using algal chloroplasts as novel bioreactors for the production of many other biologically active mammalian therapeutic proteins.

## Background

In recent years, there has been increased interest in using genetically engineered plants as an alternative expression system for the production of recombinant pharmaceutical proteins [1,2]. Plant systems offer advantages over conventional expression platforms in a number of areas, including low production cost, easy and quick scale-up, low risk of product contamination by mammalian viruses or blood-borne pathogens, and an overall higher quality of products. To date, nuclear transformed plants have been shown to be able to produce numerous recombinant proteins of therapeutic value, including human diagnostic and therapeutic full-length and single-chain antibodies, antigens, cytokines and autoantigens. Moreover, crop plants can be used for the production and delivery of safe and effective edible vaccines against various infectious and immune-related diseases (For more information, see recent reviews by Ma et al. [3,4]. Despite this promise, nuclear transformed transgenic plants often yield relatively low levels of recombinant protein. For example, the nuclear expression of hepatitis B virus (HBV) envelop surface protein in transgenic tobacco plants was reported as 0.01% of total soluble protein (TSP) [5], whereas the accumulation level of cholera toxin B subunit (CTB), a vaccine antigen against cholera, in nuclear transgenic tobacco was between 0.02 to 0.1% of TSP [6,7]. Therefore, new strategies need to be developed to overcome limited recombinant protein accumulation before the potential of transgenic plants for therapeutic protein production can be fully realized.

An alternative strategy for improving foreign protein production yield is through chloroplast transformation of higher plants or closely related eukaryotic green algae. Evidence suggests that use of transgenic chloroplasts as bioreactors offers significant advantages over nuclear transformed plants. These include high-level protein accumulation due to increased foreign gene content in chloroplasts (up to 10,000 copies/leaf cell in tobacco; or 80 copies/cell in *Chlamydomonas reinhardtii*), expression of multiple genes through a single transformation event, increased transgene containment because of maternal plastid inheritance, as well as a lack of position effects on foreign genes [8]. Additionally, the endogenous presence of chloroplast chaperones and enzymes aids in complex multi-subunit protein assembly and can correctly fold proteins containing disulfide bonds, thereby drastically reducing the costs of *in vitro *processing. High levels of foreign proteins have been obtained via expression through the chloroplast genome. For example, the expression level of CTB in chloroplast transgenic plants reached up to 4.1% of TSP [9], while its expression level in nuclear transgenic plants accounted for 0.02 to 0.1% of TSP [6,7]. Similarly, while the expression level of human serum albumin, an important therapeutic protein with many applications, in nuclear transgenic plants was around 0.2% of TSP [10], expression levels of up to 11.2% of TSP were observed in chloroplast transgenic plants [11]. There are many other vaccine antigens or biopharmaceutical proteins that have been produced in chloroplast transgenic plants. They include, for example, *Bacillus anthracis *protective antigen (PA) against anthrax [12,13], fragment C of tetanus toxin (TetC) for tetanus [14], the outer surface protein A (OspA) of *Borrelia burgdorferi *against Lyme disease [15] and cytokines such as interferonα2b (IFNα2b) and IFN-γ [16,17] as well as a diabetes-associated autoantigen human proinsulin [18]. Furthermore, many of them have been shown to be fully functional in animal studies. The reader is referred to the recent reviews by Daniell and colleagues for further information [8,19,20].

Compared to chloroplast transgenic plants, the use of chloroplast transgenic algae as a bioreactor offers several additional advantages. Microalgae, such as *C. reinhardtii*, grow and reproduce faster than any other terrestrial or aquatic plant, doubling its biomass in approximately 8 hour, and microalgae are non-toxic and non-polluting, thus environmentally friendly for mass cultivation and commercial exploitation. Also, there will be a significant reduction in the time required to generate transgenic algae as compared to higher plants. In general, stable transplastomic lines can be obtained in as little as 3 weeks, with the potential to scale up to mass production in an additional 4–6 weeks [21]. All of these have made microalgal chloroplasts to be another valuable platform for the molecular farming of pharmaceutical proteins. Indeed, the *C. reinhardtii *chloroplast expression of a large single-chain antibody has shown accumulation levels of 0.5 to 1% of algal TSP [22]. Recently, Manuell et al. [23] demonstrated robust expression of a bioactive mammalian peptide, bovine mammary-associated serum amyloid (M-SAA), in *C. reinhardtii *chloroplasts with levels up to 5% of TSP. There are several other antigenic proteins that have been produced using this system, including foot-and-mouth disease virus VP1 protein [24], tumor necrosis factor-related apoptosis-inducing ligand (TRAIL) [25] and the protein E2 of classical swine fever virus [26]. The reader is referred to recent reviews on this area for further information [21,27].

Glutamic acid decarboxylase-65 (GAD65) catalyzes the formation of gamma-aminobutyric acid (GABA) from glutamine. It is one of the major autoantigens in type 1 (insulin-dependent) diabetes, an autoimmune disease resulting from the destruction of insulin-producing β cells in the pancreas [28,29]. It has been demonstrated that many new-onset type 1 diabetic patients have autoantibodies against GAD65, with the presence of anti-GAD65 antibodies now serving as an important marker for the prediction and diagnosis of type 1 diabetes [30-32]. The identification of GAD as a major autoantigen in type 1 diabetes may also present unique opportunities for the development of novel preventative therapies against the disease. Indeed, immunization of young non-obese diabetic (NOD) mice, an animal model for human type 1 diabetes, with GAD65 or GAD peptides prevents or delays the onset of diabetes [33-35]. Furthermore, the suppression of GAD in NOD mouse islets was shown to protect the mice from developing diabetes [36]. All of these results suggest the potential importance of GAD65 in diagnosing and treating type 1 diabetes in humans. However, recombinant production of hGAD65 using conventional bacterial or mammalian cell culture-based expression systems is limited by high cost, low efficiency and low yield. To overcome these limitations, we have recently explored transgenic plants as an alternative expression platform for the production of hGAD65 [37]. Although transgenic plants offer several production advantages, including the possibility of allowing direct oral delivery of plant-derived GAD65 to induce oral immune tolerance, plant expression of hGAD65 is still limited by low accumulation levels (0.04% of TSP in tobacco).

The goal of the present study was to investigate the feasibility of using *C. reinhardtii *chloroplasts as a novel expression platform for the production of hGAD65. To this end, a chloroplast transformation vector containing the full-length *hGAD65 *gene, under the control of the *C. reinhardtii *chloroplast *rbc*L promoter as well as *rbc*L 5'- and 3'-UTRs, was generated and introduced into the chloroplast genome of *C. reinhardtii*. Here, we show that chloroplast transformed *C*. *reinhardtii *cells express and accumulate recombinant hGAD65 at levels of 0.25–0.3 % of algal TSP. Immunological analysis shows algal-derived recombinant hGAD65 reacts with Type 1 diabetic sera from NOD mice, and stimulates the proliferation of spleen lymphocytes from NOD mice. These results demonstrate that agal-derived GAD65 contains its authentic antigenicity, further suggesting the potential use for microalgae as a novel production system for human therapeutic proteins.

## Methods

### Strains, growth media and culture conditions

*C. reinhardtii *wild-type strain 137c was used as a host for chloroplast transformation. Cells of the strain 137c were maintained on Tris Acetate Phosphate (TAP) agar or grown in liquid TAP medium at 23°C under constant illumination of ~100 μE/m^2^·sec^-1^. When grown in TAP liquid medium, algal cells were cultured in flasks rotating at 100 rpm.

### Construction of the chloroplast expression vector pXW-GAD-His

To construct chloroplast expression vector pXW-GAD65-6 × His, *hGAD65 *cDNA was amplified by PCR from plasmid vector pTRL-GAD65 [37] using the primer pairs: 5'- TTCCATGGCATCTCCGGGCTCTGGC-3' (forward) and 5'-ATAATCTAGA**TTA***ATGATGATGATGATGATG*TAAATCTTGTCCAAGGCG TTC-3' (reverse). The forward primer contains an engineered *Nco*I site (underlined), whereas the reverse primer contains an *Xba*I site (underlined) immediately downstream of sequence encoding the 6 × His-tag (italic) and stop codon (bold). PCR was performed on a Perkin-Elmer Model 9600 thermocycler under the following conditions: initial denaturation for 5 min at 94°C, followed by 30 cycles of denaturation at 94°C for 30 s, annealing at 50°C for 30 s, and extension at 72°C for 60 s, followed by a final extension of 10 min at 72°C. The PCR product was isolated and blunt-end ligated into the *Sma*I site of pUC19. After verification by sequence analysis, the hGAD65 gene was released by digestion with *Nco*I and *Xba*I, blunt-ended with Klenow fragment, and cloned into *Chlamydomonas *chloroplast transformation vector pUC7-463, composed of the *rbc*L gene promoter and its 5' untranslated region (5' UTR) and 3' UTR. The resulting *hGAD65 *expression cassette was then isolated as a single *Bam*HI fragment and ligated into the *C. reinhardtii *chloroplast transformation vector p322, containing the 5.7 kb *Eco*RI/*Xho*I restriction fragment from the *C. reinhardtii *inverted repeat region (Chlamydomonas Stock Center), forming plasmid pXW-GAD-His.

### Chloroplast tranformation

*C. reinhardtii *wild type strain 137c was grown in TAP liquid medium to late log phase (approximately 7 days), with subsequent cell harvesting by centrifugation (2060 g for 10 minutes at 4°C). The cell pellet was resuspended in TAP to a density of approximately 1.0 × 10^8 ^cells/mL. Of this cell suspension, 250 μl was spotted onto the central area (1.5 cm in diameter) of a TAP agar plate and incubated in the dark at room temperature for 2 hours. After incubation, plates were bombarded with 5 μg of pXW-GAD-His DNA mixed with equal amounts of plasmid p228 DNA and coated onto tungsten particles for delivery using a Biolistic PDS-1000/He Particle Delivery System (Bio-Rad Laboratories) as described by Boynton et al. [38]. Plasmid p228, containing the 16S rRNA gene conferring spectinomycin resistance, was used to screen and/or identify transformed algal cells. Bombarded cells were incubated overnight in the dark at room temperature, re-plated onto TAP agar plates containing spectinomycin (150 μg/mL) and incubated under dim light. Colonies appearing after 2–3 weeks were re-streaked onto TAP agar plates containing spectinomycin and grown for approximately one more week. Colony cells were subcultured into TAP liquid medium containing 50 μg/ml spectinomycin and grown for one day under shaking conditions. Cells were then diluted and plated onto TAP agar plates containing spectinomycin to obtain single colonies. Several rounds of replating on selective medium were required to obtain homoplasmic cell lines.

### DNA isolation and PCR analysis

**Figure 1:**
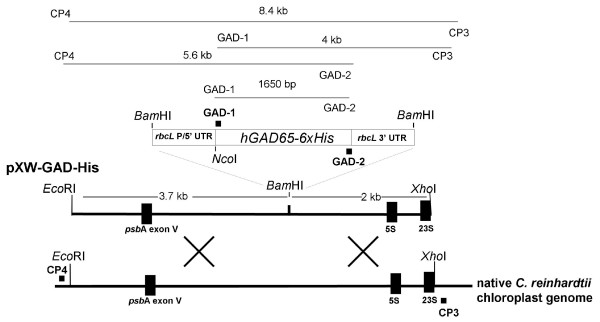
**Schematic diagram of the *hGAD65 *chloroplast expression vector pXW-GAD-His and the site of integration of the transgene cassette into the chloroplast genome**. The *hGAD65 *expression cassette consists of the *C. reinhardtii *chloroplast *rbc*L promoter and 5'UTR upstream of the transgene followed by the *rbc*L 3'UTR. The transgene cassette was inserted into plasmid vector p322 that contains a cloned 5.7 kb (*Eco*RI/*Xho*I fragment) inverted repeat of *C. reinhardtii *chloroplast DNA, resulting in pXW-GAD-His. The restriction sites used for cloning are indicated. Primers GAD-1/GAD-2 corresponding to the 5' and 3' ends of *hGAD*65 were used for PCR analysis to confirm the presence of the transgene in transformants, with the expected product size indicated. Primers CP3/CP4 complementary to sequences lying just outside the inverted repeat region of the *C. reinhardtii *chloroplast DNA were used to determine the site-specific integration of the transgene cassette into the *C. reinhardtii *chloroplast genome by PCR. The site-specific integration of the transgene cassette was additionally determined by PCR using GAD-1/CP3 and CP4/GAD-2 primer pairs. The regions for homologous recombination are indicated by the crosses. Selection of *C. reinhardtii *transformants was based on resistance to spectinomycin that was provided by co-transformation with plasmid p228 that contains the 16S rRNA gene conferring spectinomycin resistance [38].

Total DNA was isolated from wild-type *C. reinhardtii *and transformants using the method described by Newman et al. [39] with minor modifications. Briefly, cells were grown in liquid TAP medium, harvested by centrifugation (2000 × g for 10 min at 4°C) and resuspended in TEN buffer (10 mM Tris-HCl, 10 mM EDTA, 150 mM NaCl, pH 8.0). The cell suspension was centrifuged, and the pellet resuspended in 150 μl H_2_O on ice and to it, 300 μl of SDS-EB buffer (2% SDS, 400 mM NaCl, 40 mM EDTA, 100 mM Tris-HCl, pH 8.0) was added. The suspension was extracted once with 350 μl of phenol/CIA (25:24:1 by volume, phenol:chloroform:isoamyl alcohol) and the aqueous phase was collected and added with 300 μl of CIA. After a final centrifugation, the aqueous phase was collected, mixed with two volumes of 100% ethanol and incubated on ice for 30 minutes. The solution was centrifuged at 12,000 × g for 10 minutes to pellet the DNA. The DNA was then subjected to PCR analysis. To confirm the presence of the *hGAD65 *gene, PCR was performed using the following pair of *hGAD65 *specific primers: forward 5'- AAGAATTCTGGCATCTCCGGGCTCTG-3' (GAD-1), and reverse 5'- AATTCTCGAGTTATAAATCTTGTCCAAGGCG-3' (GAD-2). PCR reaction conditions were as follows: initial denaturation at 94°C for 5 min, followed by 35 cycles of denaturation at 94°C for 1 min, annealing at 50°C for 30 s, and extension at 72°C for 60 s, followed by a final extension of 10 min at 72°C. To determine the specific integration site of *GAD65 *in the chloroplast genome, long range PCR was performed using the long PCR enzyme mix (Fermentas, Glen Burnie, MD) with primer sets CP3, GAD-1 and CP4, GAD-2. The primer CP3 (5'-CCGTTCGTGCTGTGCTAGACAG-3') represents a location at one end of the inverted region of the chloroplast genome in *C. reinhardtii*, whereas the primer CP4 (5'-CGAATAACTGGGTGAATTGTCAGG-3') represents a location at the other end of this inverted region (Figure 1). PCR reaction conditions were as follows: initial denaturation at 94°C for 2 min, 10 cycles of 20 s each at 94°C, 30 s at 59°C and 4 min at 68°C followed by 25 cycles of 20 s each at 94°C, 30 s at 59°C and 4 min and 2 s at 68°C. To identify homoplasmic cell lines, PCR was performed with chloroplast specific primers CP3 and CP4 using the following reaction conditions: initial denaturation at 94°C for 5 min, 10 cycles of 20 s each at 94°C, 30 s at 57°C and 7 min at 68°C, and followed by 25 cycles of 20 s each at 94°C, 30 s at 57°C and 7 min and 5 s at 68°C. PCR products were analysed by agarose gel electrophoresis.

### RNA isolation and RT-PCR analysis

Total RNA was extracted from wild-type *C. reinhardtii *and transformants using the TRIzol RNA extraction kit according to the manufacturer's instructions. RNA was reverse transcribed to cDNA by SuperScript II Reverse Transcriptase (Invitrogen) according to the manufacturer's protocol. Briefly, 5 μg of total RNA, 1 μl Oligo(dT)_12–18 _(500 μg/ml), 1 μl dNTP Mix (10 mM each) and 5 μl sterile distilled water were mixed and incubated at 65°C for 5 min. Following addition of 4 μl First-Strand Buffer and 2 μl 0.1 M DTT, the reaction mixture was further incubated for 2 min at 42°C. After incubation, 1 μl of SuperScript™ II Reverse Transcriptase was added and incubated at 42°C for 50 min. The resulting cDNA was used as template for PCR, using hGAD65 specific primers. PCR reactions contained 2 μl of cDNA, 0.2 mM dNTPs, 2 μM of each primer, 1× reaction buffer, 1.5 mM MgCl_2_, and 2.5 U of *Taq *polymerase in a total volume of 50 μl. These reactions were incubated at 95°C for 5 min, followed by 30 cycles of 94°C for 1 min, 50°C for 1 min, 72°C for 2 min with a final extension of 10 min at 72°C. The PCR products were ran on a 1.5% agarose gel and compared against a DNA ladder (Life Technologies, Grand Island, NY).

### Western Blot analysis

Total crude protein was extracted from transformants and wild-type *C. reinhardtii *using the method as described by Goldschmidt-Clermont [40]. For immunoblot analysis, protein extract was boiled, separated on a 15% SDS polyacrylamide gel and blotted onto PVDF (polyvinylidene difluoride) membrane (Millipore, Burlington, MA). Membranes were blocked in 5% skim milk-TBST(20 mM Tris, 150 mM NaCl, 0.02% Tween 20, pH 7.6), washed with TBST, and then incubated for 1 h with a 1:2000 dilution of a rabbit anti-GAD65/67 primary antibody (Sigma-Aldrich Canada, Oakville, Ontario) followed by incubation with 1:2500 diluted horseradish peroxidase conjugated goat anti-rabbit secondary antibody. Immunodetection was performed using the enhanced chemiluminescence (ECL) detection system (Perkin Elmer Life Sciences, Rockford, IL) according to the manufacturer's instructions. Quantification of the expression level of hGAD65 in algal cells was performed by a sandwich ELISA. In brief, a 96-well microtiter plate was coated with mouse anti-GAD65 (Abcam, Cambridge, MA) antibody at a concentration of 0.2 μg/well, and incubated at 4°C overnight. The wells were washed three times with PBST (phosphate saline containing 0.05% Tween-20), and blocked with 3% BSA in PBS for 2 hours at room temperature. After washing three times with PBST, 1 μg of extracted total algal protein was added per well, and plates incubated overnight at 4°C. After washing with PBST, 0.2 μg of rabbit anti-GAD65 (Serotec, Hornby, Canada) was added per well and incubated at room temperature for 2 hours. After washing, 50 μL of 1:2000 diluted HRP-conjugated anti-rabbit IgG antibody (Kirkegaard & Perry Laboratories, Gaithersburg, USA) was added per well and incubated at 37°C for 1 hour. After incubation, 100 μL/well of TMB substrate (R&D Systems, Minneapolis, MN) was added and incubated at 37°C for 15 minutes for color development. The color reaction was stopped by addition of 100 μL/well stop solution (R&D Systems, Minneapolis, MN). The plate was read in a microplate reader (Bio-Rad 3550) at 450 nm. The hGAD65 concentration in samples was determined by comparison to a standard curve created with purified hGAD65 standard (Diamyd Diagnostics, Sweden).

### Purification of algal-derived hGAD65 protein

Algal-derived recombinant hGAD65 was purified by histidine affinity chromatography using HiTrap Chelating HP columns (GE Healthcare) according to the manufacturer's instructions. In brief, a total of 100 ml of *C. reinhardtii *cells were homogenized in 1 ml extraction buffer (750 mM Tris-HCl, pH 8.0; 15% sucrose; 100 mM β-mercaptoenthanol; 1 mM PMSF). The homogenate was centrifuged at 13,000 × g for 20 min at 4°C. The supernatant was filtered through a 0.45 μm membrane filter, and loaded onto a HiTrap Chelating HP column and washed with wash buffer (10 mM imidazol, 20 mM Na_2_HPO_4_, 500 mM NaCl) to remove nonspecifically bound endogenous algal proteins. The bound algal-derived hGAD65 was eluted with elution buffer (500 mM imidazol, 20 mM Na_2_HPO_4_, 500 mM NaCl). Fractions were collected and analysed by SDS-PAGE and ELISA. The hGAD65 fraction was then dialyzed extensively against PBS to remove high salt and imidazolel, and concentrated using a speed vacuum.

### Determination of immunoreactivity of algal-derived hGAD65 with diabetic sera by ELISA

The antigenicity of algal-derived hGAD65 was determined by its immunoreactivity with diabetic sera from NOD mice using ELISA. In brief, purified algal-derived recombinant hGAD65 was added to a 96-well microtiter plate in a volume of 50 μl/well (10 μg GAD65/ml diluted in 0.1 M NaHCO_3_, pH 8.5) and incubated overnight at 4°C. After incubation, the plate was washed, blocked with 3% BSA in PBS solution, and incubated overnight at 4°C with diabetic and control serum samples (50 μl/well in triplicate). Diabetic serum samples were obtained from newly-onset diabetic NOD mice, whereas control serum samples were collected from BALB/c mice. After washing, 50 μL of 1:2000 diluted HRP-conjugated anti-mouse IgG antibody was added per well and the plate incubated at 37°C for 2 hours. The color development was performed as described above for sandwich ELISA.

### Spleen cell proliferation assay

For the spleen cell proliferation assay, spleen cell suspensions were prepared from 8-week-old NOD mice. In brief, individual spleens were pressed through a sterile Falcon cell strainer (Becton Dickinson, Franklin Lakes, NJ), and lysed with ammonium chloride potassium (ACK) lysis buffer (Gibco/BRL, Rockville, MD) to remove erythrocytes. The resulting cell suspension was washed with DPBS (Dulbecco's phosphate buffered saline, Sigma-Aldrich Canada, Oakville, Ontario), and resuspended in complete RPMI 1640 media at a viable cell concentration of 5 × 10^5 ^ml^-1^. Spleen cells were plated in triplicate in 96-well flat-bottom microtitre plates (100 μl/well), with insect cell-derived hGAD65 standard (Diamyd Diagnostics, Sweden), algal-derived hGAD65 or the unrelated protein antigen ovalbumin (OVA) (100 μl/well of 10 μg/ml hGAD65 or OVA) added. The cell cultures were incubated at 37°C in a 5% CO_2_-humidified incubator for 72 hours and pulsed with 1 μCi/well tritiated thymidine. After a 16 hour post growth, cells were harvested, and the tritiated thymidine incorporated was determined by using a Beckman LS 6500 liquid scintillation counter (Beckman Coulter, Fullerton, Califonia). Results were shown as the mean counts per minute (CPM) of triplicate spleen-cell cultures.

## Results

### Construction of a chloroplast expression vector and algal transformation

The plasmid pXW-GAD-His (Figure 1) was constructed and used for *C. reinhardtii *chloroplast transformation. The expression of hGAD65 was under the control of the *C. reinhardtii *chloroplast *rbc*L promoter and 5' UTR and 3' UTR. A C-terminal 6 × His tag was added to facilitate purification of the recombinant protein using metal-affinity chromatography. The chimeric gene was flanked by sequence from an inverted repeat region of the *C. reinhardtii *chloroplast genome to provide sites for *in vivo *homologous recombination. The plasmid pXW-GAD-His was transformed into wild-type *C. reinhardtii *chloroplasts by particle bombardment along with the plasmid p228 conferring spectinomycin resistance as a means of selection.

### PCR analysis of *C. reinhardtii *transformants

**Figure 2:**
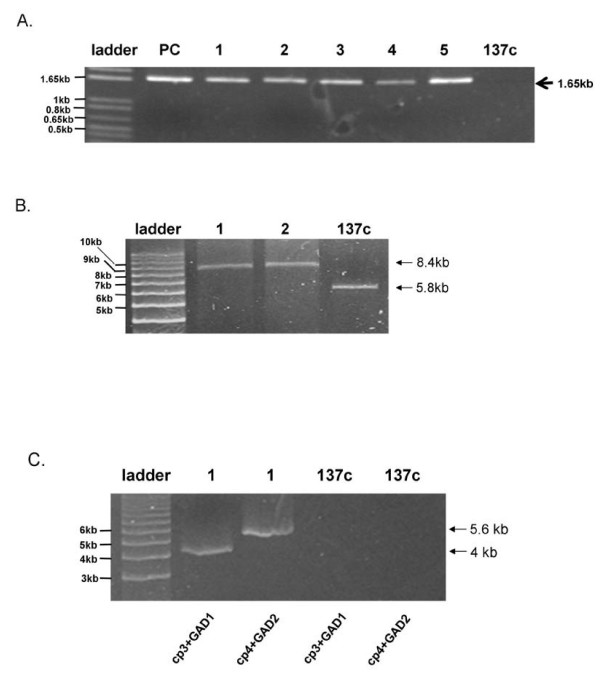
**PCR analysis of *C. reinhardtii *transformants**. (A) hGAD65 specific primers GAD-1 and GAD-2 generated PCR products using total algal DNA as template. Lanes 1 to 5, independent *C. reinhardtii *transformants; PC, positive control (*hGAD65 *containing plasmid pXW-GAD-His). *C. reinhardtii *wild-type strain 137c DNA was used as a negative control. PCR products are indicated by the arrow. (B) Chloroplast specific primers CP3 and CP4 generated PCR products using total algal DNA as a template. Lanes 1 to 2, representative *C. reinhardtii *transformants; *C. reinhardtii *wild-type strain 137c DNA was used as a negative control. PCR products are indicated by the arrows. (C) The CP3/GAD-1 or CP4/GAD-2 primer set generated PCR products. Lane 1, representative *C. reinhardtii *transformant. *C. reinhardtii *wild-type strain 137c DNA was used as a negative control. PCR products are indicated by the arrows.

*C. reinhardtii *transformants were selected on TAP agar plates supplemented with spectinomycin (TAP-Spec). Primary putative transformants were initially screened by PCR for the presence of the *hGAD65 *gene using *GAD *specific primers. As shown in Figure 2A, PCR amplification of the genomic DNA obtained from primary transformants resulted in the synthesis of a product with expected 1.65 kb size. No PCR products were produced from DNA isolated from wild-type *C. reinhardtii*. Positive transformants were administered additional rounds of selection in order to obtain homoplasmic cell lines in which all copies of the chloroplast genome contained the introduced chimeric *hGAD65 *gene. To demonstrate that the *hGAD65 *expression cassette was site-specifically integrated into the algal chloroplast genome, long PCR amplification of genomic DNA from selected transformants was performed using a combination of primer sets. As indicated in Figure 1, primers CP3 and CP4 were designed to be complementary to sequences lying just outside the inverted repeat region of the *C. reinhardtii *chloroplast, and therefore PCR amplification of genomic DNA from transformants with a CP3 and CP4 primer pair would give an expected 8.4 kb DNA product if the *hGAD65 *cassette was correctly inserted into the inverted region of the algal chloroplast genome. As shown in Figure 2B, a DNA fragment of expected size was produced after PCR amplification. In contrast, PCR amplification of genomic DNA from wild-type *C. reinhardtii *using the same CP3/CP4 primer pair produced a band of smaller molecular weight (5.8 kb), equivalent in size to the inverted repeat region of the algal chloroplast DNA lacking inserted foreign DNA. The apparent lack of amplification of any 5.8 kb product from the inverted repeat region of chloroplast DNA with transformants further implies that transformants are all homoplasmic. Additional long PCR was performed using a combination of GAD and chloroplast specific primers to show foreign DNA inserted to the chloroplast inverted region was indeed that of *hGAD65*. The primer GAD-1 was designed according to the N-terminal coding sequence of hGAD65. When CP3 and GAD-1 were used as a primer pair, a 4-kb PCR product spanning the full hGAD65 coding sequence (1.65 kb), 3' *rbc*L UTR (440 bp) and the *Xho*I-*Bam*HI fragment (2 kb) of the flanking inverted repeat region would be expected. As shown in Figure 2C, a 4-kb DNA fragment was obtained with the use of this primer pair. Similarly, when GAD-2 (designed for the C-terminal coding sequence of hGAD65) and CP4 were used as a primer pair, an expected 5.6-kb PCR product (corresponding to the full hGAD65 coding sequence (1.65 kb), chloroplast *rbc*L promoter and 5' UTR (390 bp) and a 3.7-kb *Bam*HI-*Eco*RI fragment of the flanking inverted repeat region) was amplified from the same transformant (Figure 2C). As expected, no PCR products were generated from *C. reinhardtii *wild-type strain 137c DNA when the same CP3 and GAD-1 or CP4 and GAD-2 primer pair was used (Figure 2C). Taken together, these results suggest all transformants analysed were homoplasmic for the *hGAD65 *cassette site-specifically inserted into the host chloroplast genome.

### Transcriptional analysis of hGAD65 in *C. reinhardtii *transformants by RT-PCR

**Figure 3:**
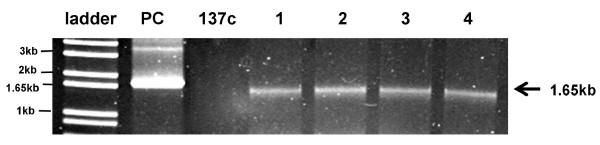
**RT-PCR detection of *hGAD65 *mRNA expression in *C. reinhardtii *transformants**. Total RNA was isolated from *C. reinhardtii *wild-type strain 137c and transformants. RNA was transcribed to cDNA as described in Methods. Resulting cDNA was amplified using primers specific to *hGAD65*. Lanes 1 to 4, PCR amplification of cDNA from independent transformants. PCR amplification of cDNA from wild-type strain 137c was used as a negative control. PC, positive control (direct PCR amplification of DNA from plasmid pXW-GAD-His). PCR products are indicated by the arrow.

The transcriptional expression of *hGAD65 *in *C. reinhardtii *transformants was evaluated by RT-PCR from total RNA isolated from wild-type *C. reinhardtii *and transformants. When *hGAD65 *specific primers were used, a product of expected size (1.65 kb) was detected in cDNA reverse-transcribed from RNA isolated from selected transformants (Figure 3). No RT-PCR products were detected in cDNA from wild-type *C. reinhardtii*. To rule out possible amplification of contaminant DNA in the samples, direct PCR amplification without reverse transcription was performed on the RNA preparations. No amplified PCR products were seen under the same conditions, confirming the specificity of the RT-PCR reaction (data not shown). In summary, these data indicate that *hGAD65 *is actively transcribed in chloroplast transformed algal cells.

### Accumulation of hGAD65 protein in *C. reinhardtii *transformants

To demonstrate the accumulation of hGAD65 protein, total soluble protein (TSP) was prepared from wild-type *C. reinhardtii *and transformants, and analyzed by Western blotting.

**Figure 4:**
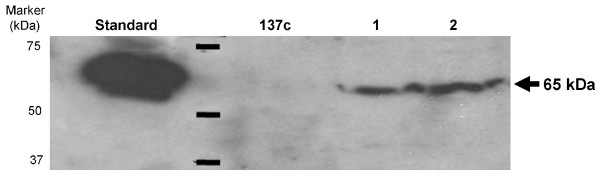
**Western blot detection of recombinant hGAD65 from total soluble protein extracted from chloroplast transgenic algal lines**. Samples containing ~50 ng of commercial hGAD65 or 5 μg of total algal protein were separated by 15% sodium dodecylsulphate-polyacrylamide gel electrophoresis (SDS-PAGE), transferred onto a polyvinylidene difluoride membrane and probed with rabbit anti-GAD65 primary antibody followed by horseradish peroxidase conjugated goat anti-rabbit secondary antibody. Standard, insect cell-derived hGAD65. Lanes 1 to 2, protein extracts from two representative algal transformants. Protein extracts from wild-type strain 137c were used as a negative control. Arrows indicate the position of algal-derived hGAD65.

**Figure 5:**
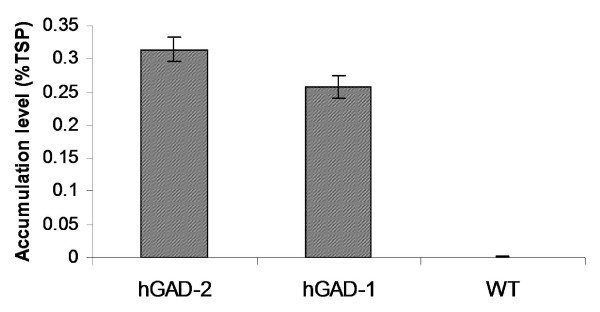
**Expression levels as percentages of hGAD65 in total soluble protein (TSP) of hGAD65 expressing *C. reinhardtii *transformants**. The expression level was determined by hGAD65 specific ELISA as described in detail in methods. hGAD-1 and hGAD-2, two independent algal transformants; WT, untransformed wild type *C. reinhardtii *137c strain.

Anti-human GAD65 polyclonal antibody detected a single 65-kDa band, corresponding in size to the hGAD65 protein (Figure 4). No protein band was detected in extracts from *C. reinhardtii *wild-type strain 137c. Accumulation levels of hGAD65 in algal cells were measured using ELISA. As shown in Figure 5, algal derived hGAD65 accounts for up to 0.25 to 0.3% of TSP.

### Immunoreactivity of algal-derived hGAD65 with sera from diabetic NOD mice

**Figure 6:**
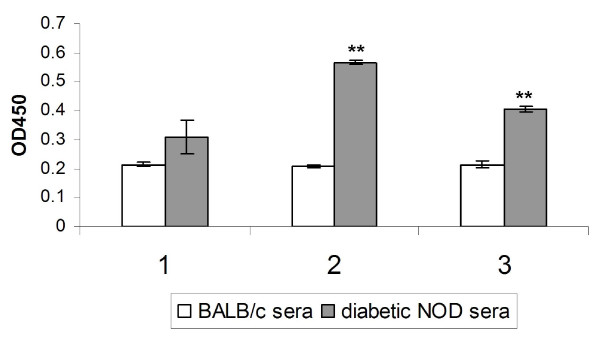
**Demonstration of immunoreactivity with NOD mouse diabetic sera to algal-derived hGAD65**. Purified algal-derived hGAD65 was coated onto 96-well plates and incubated with serum samples from diabetic NOD mice (n = 3) and reference BALB/c mice (n = 3). Bound anti-GAD antibody was detected with HRP-conjugated anti-mouse IgG. Error bars represent the standard deviation. ** above bars indicates a significant difference from reference BALB/c sera (*p *< 0.05).

Since prediabetic patients with type 1 diabetes and NOD mice contain anti-GAD antibodies in their sera [36], the antigenicity of algal-derived hGAD65 was determined by its immunoreactivity with diabetic sera. Thus, purified algal-derived hGAD65 was coated onto a microtiter plate and tested against three diabetic serum samples collected from NOD mice. Three serum samples obtained from BALB/c mice were used as non-diabetic controls. As shown in Figure 6, all three diabetic serum samples had higher binding capacity to algal-derived hGAD65 than did reference serum samples, with samples number 2 and 3 showing a significant difference (p < 0.05). These results suggest that algal-derived recombinant hGAD65 is antigenic.

### Effect of algal-derived hGAD65 on spleen cell proliferation

**Figure 7:**
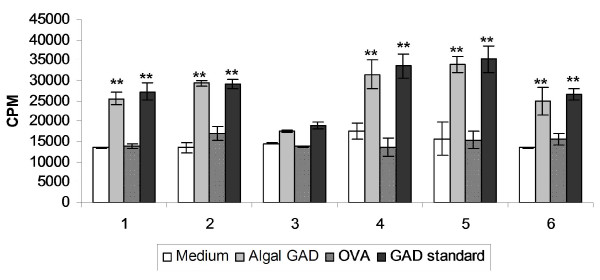
**Spleen cell proliferation responses to algal-derived hGAD65**. Individual 8-week-old NOD mice were tested for spontaneous proliferation of splenocytes to insect cell-derived hGAD65 standard, algal-derived hGAD65 or to the unrelated protein antigen ovalbumin (OVA). Error bars represent the standard deviation. ** above bars indicates a significant difference from OVA and medium alone (*p *< 0.05).

To further assess the antigenicity of algal-derived hGAD65, the effect of algal-derived hGAD65 on spleen cell proliferation was analyzed. Splenocytes were isolated from individual 8-week-old NOD mice and cultured with medium alone or stimulated with insect cell-derived hGAD65 standard, algal-derived hGAD65, or the irrelevant protein antigen ovalbumin (OVA). As shown in Figure 7, NOD mouse-derived spleen cells proliferated significantly in response to *in vitro *stimulation with algal-derived hGAD65, with a magnitude of response comparable to the insect cell-derived hGAD65 standard. In contrast, NOD mouse-derived spleen cells cultured with medium alone or stimulated with OVA showed no significant proliferative activity. The difference in cell response to algal-derived hGAD65 and OVA is significant (p < 0.05). These results provide additional evidence that algal-derived hGAD65 is indeed immunogenic.

## Discussion

The production of recombinant hGAD65 has been achieved in a number of expression systems including insect cells and *E. coli*, but all have been limited by their efficiency. Only low levels of hGAD65 accumulation was obtained in baculovirus-infected SF9 insect cells [41]. Moreover, this expression platform is technically and economically demanding, and hence is expensive and difficult to scale-up for mass production. The expression of hGAD65 as a fusion protein has been demonstrated in *E. coli *[42], but results in accumulation of recombinant product in insoluble inclusion bodies, needing solubilization, renaturation as well as chemical or enzymatic procedures to separate the target protein from its fusion partners. These additional processes can be expensive and time consuming. Our recent demonstration that hGAD65 can be expressed in transgenic tobacco may alleviate some of the problems associated with conventional expression platforms for hGAD65 production [37]. However, plant genomic expression of hGAD65 is still limited by low accumulation levels (0.04% of TSP). Here we report the use of algal chloroplasts as a new type of bioreactor for the production of hGAD65. *C. reinhardtii *transformed with the *hGAD65 *gene under control of the chloroplast *rbc*L promoter and 5'-UTR was produced. The site-specific integration of the transgene into the chloroplast genome of *C. reinhardtii *was demonstrated by PCR (Figure 2). RT-PCR analysis revealed viable expression of *hGAD65 *mRNA in *C. reinhardtii *transformants (Figure 3). The accumulation of recombinant hGAD65 protein was further confirmed by Western blotting using anti-GAD antibody (Figure 4). As measured by ELISA, *C. reinhardtii *derived hGAD65 protein accounted for up to 0.25 to 0.3% of total algal soluble protein (TSP) (Figure 5). This value is much higher when compared to hGAD65 expression in nuclear transgenic plants (0.04% TSP) [37]. With a significant increase in immunologically active hGAD65 accumulation, taken together with *C. reinhardtii*'s rapid growth rate, it is obvious that microalgae represent a superior platform for hGAD65 production. In addition, algae are one of the easiest living organisms to propagate, requiring only sunlight, carbon dioxyde and water to thrive. It can be easily grown to industrial levels at very low cost. There are several common methods that can be used to grow algae at large scale, including the use of ponds, cheap flexible plastic bags or a solar photoreactor. As only the unmodified native form of hGAD65 gene under control of the chloroplast *rbc*L gene promoter and its 5'-UTR was investigated in this pilot study, it is expected accumulation levels of hGAD65 in algal cells could be improved further if a *C. reinhardtii *chloroplast codon-optimized version of hGAD65 gene is used. The native hGAD65 DNA contains relatively low AT content (52%) and its expression may not be favored by an AT-rich *C. reinhardtii *chloroplast genome in which the overall AT content is 65.5% [43]. Franklin et al. [44] showed an 80-fold increase in expression levels of GFP (green fluorescent protein) in *C. reinhardtii *chloroplasts when a synthetic GFP gene with increased AT content (66% AT content in synthetic GFP in comparison to native GFP with 60% AT content) was used. Furthermore, as the 5*'-*UTR region of chloroplast mRNAs has a profound effect on the translational efficiency of *C. reinhardtii *chloroplast genes [45,46], the use of different 5'-UTRs of chloroplast genes such as the 5'-UTR of the plastid *psb*A gene (D1 protein of photosystem II), combined with a strong promoter, could serve as another approach to enhance hGAD65 expression in *C. reinhardtii *cells. One additional strategy to improve hGAD65 expression may be the use of different host strains of *C. reinhardtii*. Mayfield and Schultz [47] reported a much higher expression (10-fold increase) of the luciferase reporter gene in *C. reinhardtii *chloroplasts when it was expressed in a *C. reinhardtii *mutant strain that lacked the corresponding endogenous gene (ie, the *psb*A deficient strain cc744).

The antigenicity of algal-derived hGAD65 was demonstrated based on its immunoreactivity with diabetic sera as well as its ability to stimulate the *in vitro *proliferation of splenic T cells derived from NOD mice. Anti-GAD antibodies are present in >70% of newly diagnosed type 1 diabetic patients and have been detected up to 7 years before clinical onset of the disease [48,49]. Measurement of anti-GAD antibodies has been proposed as a better predictor for the future development of type 1 diabetes in people at high risk [50]. Anti-GAD antibodies are also present in the sera of a majority of NOD mice and can be detected at an early stage of the disease [33,34]. Our results indicate that algal-derived hGAD65 reacts specifically with diabetic sera (Figure 6), suggesting its authentic GAD antigenicity. The *in vitro *spleen cell proliferation assay provides further evidence that algal-derived hGAD65 is immunogenic (Figure 7). In this assay, spleens derived from 8-week-old NOD mice were used as a source of spleen cells. In NOD mice, spontaneous proliferative T cell responses to GAD65 is seen as early as 4 weeks of age, parallel to the onset of insulitis. Also, the proliferative response is initially confined to limited regions on the GAD65 molecule and subsequently spreads intramolecularly to different regions of GAD65 and intermolecularly to other β cell antigens such as insulin [51]. Kaufman et al. [34] showed that spleen cells from 8-week-old NOD mice proliferate in response to GAD peptides. This study shows that NOD mouse-derived spleen cells proliferate when stimulated with algal-derived hGAD65, and that the stimulatory activity of algal-derived hGAD65 is comparable to insect cell-derived hGAD65 standard. As expected, addition of an unrelated protein antigen (OVA) had little effect on the proliferation of NOD mouse-derived spleen cells. The proliferation of spleen cells from one mouse (mouse #3) was not significantly altered by the stimulation with either algal-derived hGAD65 or insect cell-derived hGAD65 standard. This is most likely due to a lower frequency of GAD reactive T cells within the spleen of this mouse as compared to other NOD mice. This observation is supported by Kaufman et al. [34] who examined the *in vitro *proliferative responses of spleen cells from individual NOD mice to GAD stimulation and showed variations in their proliferative responses to GAD.

The previous work by Ruhlman et al. [18] has showed the expression of diabetes-associated autoantigen human proinsulin in plant chloroplasts. In the present work, we have demonstrated the usefulness of *C. reinhardtii *chloroplasts as another platform for the production of diabetes-associated autoantigen hGAD65. To our knowledge, this is the first report on the use of algal chloroplasts for the production of a human autoantigenic protein. This demonstration opens the way for future use of *C. reinhardtii *chloroplasts as bioreactors for the production of other therapeutic proteins.

## Conclusion

Currently there are no efficient expression systems available for recombinant production of hGAD65. In the present study, we have demonstrated that transplastomic *C. reinhardtii *is a superior expression platform for the production of hGAD65. This is the first report on the use of *C. reinhardtii *chloroplasts for the production of a full-length autoantigenic protein. The ability to produce low-cost hGAD65 in large quantities will facilitate the development of immunoassays useful for screening and monitoring large numbers of individuals for susceptibility to type 1 diabetes, and also for the treatment of patients with type 1 diabetes.

## Authors' contributions

SM and NH conceived and led the project. XW constructed the vectors, and performed the *C. reinhardtii *chloroplast transformation, Western blotting analysis, algal hGAD65 protein purification and its functional assays. MB conducted transplastomic *C. reinhardtii *genomic DNA isolation, determined the integration of hGAD65 cassette into the chloroplast genome by PCR and analyzed the transcriptional expression of hGAD65 by RT-PCR. RT developed hGAD65 ELISA protocol and performed the assay to demonstrate the expression level of hGAD65 in *C. reinhardtii*. AMJ and DM participated in experimental design.
